# Sourdough-Yeast Co-Fermentation Preserves Frozen Raw Stuffed-Bun Quality

**DOI:** 10.3390/foods15173074

**Published:** 2026-08-30

**Authors:** Xiaoling Zhou, Dongsheng Zhang, Sinan Xu, Yanju Hu, Bin Xu, Zhihua Li, Xiaobo Zou

**Affiliations:** 1 School of Food Science and Engineering, Jiangsu University, Zhenjiang 212013, China; 15111312291@126.com (X.Z.);; 2 Chen Ke Ming Food Manufacturing Co., Ltd., No. 1 Xing Sheng Avenue, Industrial Park, Nanxian, Yiyang 413200, China

**Keywords:** sourdough-yeast fermentation, microbial metabolism, frozen raw steamed stuffed buns, quality retention

## Abstract

Frozen raw steamed stuffed buns lose volume and harden during storage, limiting industrial quality retention. Here, we evaluated 0%, 30%, 50%, 70%, and 100% sourdough addition in a sourdough–yeast co-fermentation system and examined microbial, metabolic, and gluten-related changes. At 30% addition, the dough reached a maximum height of 47.7 mm and produced the best overall product quality. After 30 days at −18 °C, this group retained the highest specific volume, approximately 21% above the control, together with lower hardness and higher sensory scores. Yeast and lactic acid bacteria (LAB) counts remained at 6.21 and 7.50 lg CFU/g, respectively, and proline accumulation was positively correlated with greater freeze tolerance, suggesting that it may play a role in cryoprotection, but the causal relationship remains to be further experimentally verified. Free sulfhydryl content increased by 6.25%, compared with 73.02% in the control, indicating less disruption of gluten disulfide bonds. Therefore, under the experimental conditions employed in this study (specific wheat flour, sourdough fermentation system, and 30-day frozen storage), the 30% sourdough addition level exhibited the best overall quality improvement, probably through coordinated microbial metabolism and preservation of the gluten network. These findings support the use of sourdough–yeast co-fermentation in frozen flour products.

## 1. Introduction

Demand for frozen flour-based foods is increasing because they combine convenience, long shelf life, and standardised production [1]. Frozen raw steamed stuffed buns can retain characteristics of freshly prepared products while reducing final processing. Frozen raw steamed stuffed buns are defined as buns that are rapidly frozen immediately after molding, either without proofing or without steaming after proofing. They are maintained under frozen conditions during transport and storage. For consumption, they may be steamed directly without thawing or after a short thawing/proofing step. Given the increasing demand for convenience foods and the extended shelf life achieved by freezing, their commercial importance has grown considerably. However, frozen storage and subsequent steaming reduce specific volume, increase hardness, and impair elasticity and flavour [2,3,4]. Ice-crystal formation and recrystallisation damage the gluten network and reduce yeast viability [5,6,7,8]. Starter selection is therefore central to frozen-dough stability. Industrial production relies mainly on commercial *Saccharomyces cerevisiae* for rapid, controllable gas production. However, yeast-only products may stale rapidly and lack the flavour and texture associated with traditional starters [9,10]. Sourdough comprises flour, water, LAB, yeasts, and other fungi, with LAB usually dominant [11]. LAB-mediated fermentation can delay staling, improve nutritional properties, and extend the shelf life of steamed buns [12,13]. Multi-strain fermentation also generates esters, aldehydes, alcohols, and organic acids that shape flavour and texture [14]. Nevertheless, traditional sourdough is difficult to standardise because fermentation is slow and sensitive to raw materials and environmental conditions. Combining sourdough with commercial yeast could couple rapid gas production with microbial metabolites that influence gluten structure, flavour, and freeze tolerance. Despite previous studies exploring the application of sourdough in frozen dough systems, most have focused on Western-style products such as bread, and systematic research on sourdough–yeast co-fermentation in frozen raw steamed stuffed buns—a specific Chinese pastry product—remains limited. Among the existing reports, some studies have employed single dominant lactic acid bacteria strains (e.g., *Lactobacillus plantarum*, *Lactobacillus fermentum*, etc.) isolated from traditional sourdoughs for fermentation [15,16]. However, single-strain fermentation often exhibits lower metabolite diversity and limited gluten network modification capacity compared to multi-strain co-culture systems [17]. In contrast, although traditional sourdoughs containing complex microbial consortia possess high microbial diversity, their intricate community structure and poorly controllable fermentation behavior hinder their stable application in industrial production. Moreover, existing studies have generally adopted a single addition level or limited gradients (e.g., simply comparing addition versus non-addition), lacking a systematic evaluation of the dosage effects of different sourdough addition levels, nor have they deeply explored how sourdough addition modulates the microbial community structure and metabolic profiles to subsequently affect the dough protein network and the frozen storage quality of the final products. We therefore tested five sourdough addition levels (0–100%) and measured fermentation, colour, specific volume, texture, sensory quality, microbial communities, metabolites, and gluten-protein changes during frozen storage. The study aimed to identify an addition level that balances initial product quality with storage stability and to explore possible associations between microorganisms and their metabolites and dough properties.

## 2. Materials and Methods

### 2.1. Materials

A traditional natural sourdough starter, which is a spontaneously fermented culture containing a mixed population of lactic acid bacteria and yeasts, was obtained from Zhumadian, Henan Province, China. Sourdough was prepared using flour, starter, and water at 50:10:32 (*w*/*w*/*w*), yielding a honeycomb structure and pH 4.6–4.8. For propagation, flour, sourdough, and water were mixed at 200:40:100 (*w*/*w*/*w*) and fermented at 4–7 °C for approximately 6–8 days until the pH reached 5.3–5.4. The sourdough was stored at 4 °C and refreshed every 7 days using this procedure. Commercial yeast and edible alkali were purchased from Angel Yeast Co., Ltd., Yichang, China; wheat flour from Chen Ke Ming Food Manufacturing Co., Ltd., Yiyang, China; and white sugar from Guangxi Boqing Food Co., Ltd., Hechi, China.

### 2.2. Sample Preparation and Formulation Design

#### 2.2.1. Determination of Dough Formulations with Varying Sourdough Addition Levels

Five sourdough addition levels (0%, 30%, 50%, 70%, and 100%) were defined relative to total flour, comprising wheat flour and the flour contributed by the sourdough. Wheat flour and added water were adjusted to maintain constant total flour and comparable dough consistency. Food-grade alkali was then added to adjust the initial dough pH to 6.3–6.5, as measured by a pH meter (a-AB33M1 ZH, OHAUS Instruments Co., Ltd., Changzhou, China). The formulations are listed in Table 1.

#### 2.2.2. Preparation of Frozen Raw Steamed Stuffed Buns

Frozen raw steamed stuffed buns were prepared as follows: wheat flour, sourdough starter, yeast, white sugar, edible alkali, and water were mixed according to the specified formulation. The ingredients were mixed at 20 °C for 4 min using a stirrer (SM2-10, Xinmai Machinery Co., Ltd., Wuxi, China) to form a uniform dough. The dough was sheeted to the required thickness using a noodle pressing machine (YQ-Y30, Junling Kitchenware Co., Ltd., Shunde, China) and then rolled into wrappers. Mushroom and green vegetable filling was placed proportionally on the dough wrappers, and the filled wrappers were molded into steamed stuffed buns. Each bun was 70 ± 0.5 g and of uniform size. The wrapper:filling ratio was 42:28 (*w*/*w*), and this ratio and the filling formulation were consistent across all groups. The shaped buns were proofed in a Proofer (K12-1086D 16G, Gaobi Baking Equipment Co., Ltd., Huizhou, China) at 38 °C and 85% relative humidity for 30 min, followed by resting at ambient temperature for 5 min for cooling. The ultra-low temperature freezer (DJL-QF-30A, Dejieli Cryogenic Technology Co., Ltd., Shenzhen, China) was pre-cooled to −80 °C for 4 min prior to use. The stuffed buns were then placed into the pre-cooled freezer and rapidly frozen at −80 °C for 16 min to quickly pass through the maximum ice crystal formation zone (−1 °C to −5 °C), thereby inhibiting the formation of large ice crystals and minimizing damage to the gluten network and yeast cells. Immediately after pre-freezing, the buns were packaged and transferred to −18 °C for frozen storage for 30 days according to the experimental design.

For each treatment, three independent batches were produced on separate days (biological replicates, *n* = 3). From each batch, multiple buns were sampled for quality analyses. For measurements that involved repeated determinations on subsamples from the same batch (e.g., texture, color), these were treated as technical replicates, and the mean value from each batch was used as the experimental unit for statistical analysis. For each analysis, the specific number of buns sampled per batch and the number of technical replicates are indicated in the corresponding subsections. Data were expressed as mean ± standard deviation (SD).

### 2.3. Determination of Dough Fermentation Rheological Properties

The fermentation characteristics of the dough were determined using a fermentograph (F4, Chopin Technologies, Villeneuve-la-Garenne, France) according to the method described by Jiang et al., with minor modifications [18]. Fresh, non-frozen dough (300 g), prepared as described in Section 2.2.2, was weighed and placed into the dedicated fermentation basket of the instrument. Before testing, the fermentograph was preheated until the chamber temperature stabilised at 38 °C.

The test was run for 3 h under a 2 kg load. Maximum dough height (H_m_), time to gas leakage (T_x_), total gas production (V_t_), and gas-retention coefficient (R_c_) were recorded. Fermentograph measurements were performed in 2 technical replicates.

### 2.4. Quality Parameter Evaluation of Frozen Raw Steamed Stuffed Buns During Storage

Frozen raw steamed stuffed buns were steamed in a steam oven (LM100BE, Rational AG, Landsberg am Lech, Germany) over high heat (100 °C) for 17 min without thawing and cooled at 25 °C for 30 min before analysis.

#### 2.4.1. Color Measurement

Crust colour (L*, a*, and b*) was measured at three points using a CR-400 colorimeter. Crust color (L, a, b*) was measured using a CR-400 colorimeter (Konica Minolta Co., Ltd., Tokyo, Japan) at three points on each of three buns per treatment group. The whiteness index (WI) was calculated according to Tuncel et al. [19], as shown in Equation (1):

(1) $$WI=100-\sqrt{{\left(100-L^{*}\right)}^{2}+{a^{*}}^{2}+{b^{*}}^{2}}$$

where L* denotes lightness, a* the red–green component, and b* the yellow–blue component.

#### 2.4.2. Determination of Specific Volume

Specific volume was measured using a volumetric analyser (BVM6600, Perten Instruments, Stockholm, Sweden) with an elliptical reference shape at 1 rps and calculated as follows:

Specific volume (mL/g) = Volume (mL)/Mass (g)(2)

Specific volume was determined in 3 technical replicates.

#### 2.4.3. Analysis of Textural Properties

The middle section of the bun skin was cut into 2 cm × 2 cm × 1 cm pieces for texture-profile analysis using a TVT 6700 analyser (Perten Instruments, Stockholm, Sweden) with a P35 probe. The test speed was 1 mm/s, deformation was 50%, and trigger force was 5 N. Five technical replicates were conducted for each sample.

#### 2.4.4. Sensory Assessment of Frozen Raw Steamed Stuffed Buns

Sensory quality was assessed by ten trained panelists following the Yangzhou Culinary Association standard T/YZPX003-2016, with minor modifications (Table 2), on frozen raw steamed stuffed buns stored for 1, 15, and 30 days.

### 2.5. Microbial Community and Viable Cell Count Analysis

#### 2.5.1. Determination of Microbial Diversity

Total DNA was extracted using a HiPure Soil DNA Kit (Guangzhou Magen Biotechnology Co., Ltd., Guangzhou, China), and the concentration and purity were determined using a NanoDrop spectrophotometer (Thermo Fisher Scientific, Wilmington, DE, USA). Bacterial amplification was performed using the primers 341F (5′-CCTACGGGNGGCWGCAG-3′) and 806R (5′-GGACTACHVGGGTATCTAAT-3′), while fungal amplification was conducted using the primers ITS3_KYO2 (5′-GATGAAGAACGYAGYRAA-3′) and ITS4 (5′-TCCTCCGCTTATTGATATGC-3′). Sequencing libraries were constructed using the Illumina DNA Prep Kit (Illumina, CA, USA). Library quality was assessed using the ABI StepOnePlus Real-Time PCR System (Life Technologies, Foster City, CA, USA). Qualified libraries were pooled and sequenced on the NovaSeq 6000 platform using the PE250 mode. Raw reads were filtered using FASTP (version 0.18.0), and the resulting clean reads were used for downstream analysis. Primer sequences were removed from the filtered high-quality sequences using the DADA2 package (version 1.12.1), which outputs a list of unique sequences with their abundance and the positional quality scores for each unique sequence; these scores were used to build an error model. DADA2 then applied a machine-learning-based parametric error model for denoising (i.e., sequence correction), extracted core sequences, and generated the initial amplicon sequence variants (ASVs). Chimeric tags were detected and removed using the UCHIME algorithm. The effective tags obtained after chimera filtering were used for ASV abundance statistics and other downstream analyses. Three independent biological replicates from each treatment group (originating from three independently prepared batches) were subjected to DNA extraction. Each biological replicate was independently constructed into a sequencing library and individually loaded for sequencing.

#### 2.5.2. LAB Count

Under aseptic conditions, dough was serially diluted using sterile physiological saline. Appropriate dilutions were selected and inoculated onto MRS agar plates using either the pour plate or spread plate method. Plates were incubated anaerobically at 37 °C in a constant-temperature incubator for 48–72 h, before colony counting. The results were expressed as lg colony-forming units per gram (lg CFU/g).

#### 2.5.3. Yeast Count

Appropriate sample dilutions were inoculated onto potato dextrose agar (PDA) or rose Bengal Agar medium using either the pour plate or spread plate method. Plates were incubated at 28 °C in a constant-temperature incubator for 5 days, before colony counting. The results were expressed as lg colony-forming units per gram (lg CFU/g).

### 2.6. Determination of pH, Total Titratable Acidity (TTA), and Key Metabolites

#### 2.6.1. Determination of pH and TTA

The pH and TTA of the dough were determined according to the method described by Xie et al. [20], with minor modifications. Briefly, 10.0 g of dough containing different proportions of sourdough starter were accurately weighed and placed in a clean beaker. Then, 90 mL of distilled water was added, and the mixture was stirred at moderate speed on a magnetic stirrer for 10 min to obtain a homogeneous suspension. The pH of the suspension was measured directly using a pH meter. The pH of dough samples was measured after 1 day and 30 days of frozen storage, respectively. Three technical replicates were performed for each sample. For TTA determination, a defined mass of dough sample was accurately weighed, homogenized with a measured volume of deionized water, and titrated with 0.1 mol/L NaOH standard solution until the pH reached 8.5. TTA was expressed as the volume of NaOH consumed (mL).

#### 2.6.2. Determination of Organic Acids and Ethanol Content

The contents of lactic acid and acetic acid in the samples were determined according to the method described by Tang et al. [21], with slight modifications. Briefly, 1.0 g of sample was accurately weighed into a 50 mL centrifuge tube, followed by the addition of 20 mL of ultrapure water. The mixture was homogenized and then subjected to ultrasonication for 30 min. Subsequently, the sample was centrifuged at 4000 r/min for 5 min at 4 °C. The supernatant was diluted to 25 mL with ultrapure water. The solution was filtered through a 0.22 μm membrane prior to analysis by high-performance liquid chromatography (HPLC) (2695, Waters, Milford, MA, USA) to determine the contents of lactic acid and acetic acid. The HPLC conditions were as follows: an LP C_18_ column (4.6 mm × 250 mm, 5 μm) was used, the column temperature was at 35 ± 2 °C; UV detection was performed at 210 nm, the flow rate was 0.8 mL/min; the injection volume was 10 μL; and the mobile phase consisted of 0.1% phosphoric acid aqueous solution and acetonitrile. Ethanol content was determined by gas chromatography–mass spectrometry (GC-MS) (6890N + 5975C, Agilent, Santa Clara, CA, USA) according to the method described by Xu et al. [22]. Sample pretreatment was identical to that used for organic acid analysis. Analysis used an Agilent 6890N-5975C system equipped with an HP-FFAP column (30 m × 0.25 mm, 0.25 μm), with helium as the carrier gas. The injection volume was 1 μL. Lactic acid, acetic acid, and ethanol were determined to be 0.5, 0.6, and 8 mg/kg, respectively, and the corresponding LOQs were 1.5, 1.8, and 24 mg/kg.

### 2.7. Determination of Fermentable Sugars Content

Fermentable sugar content was determined by the HPLC method specified in the Chinese National Standard GB 5009.8-2023 (National Food Safety Standard—Determination of fructose, glucose, sucrose, maltose and lactose in foods) [23]. Sample pretreatment was identical to that described in Section 2.6.2. The chromatographic conditions were as follows: Hypersil GOLD Amino column (4.6 mm × 250 mm, 5 μm), refractive index detector, injection volume of 10 μL, mobile phase of 70% (*v*/*v*) acetonitrile, and flow rate of 1 mL/min.

### 2.8. Determination of Free Amino Acids Content

Free amino acid content was determined by HPLC. Briefly, 1.0 g of freeze-dried powder sample was mixed with 10 mL of a 5% (*w*/*w*) trichloroacetic acid solution, and subjected to ultrasonic extraction for 30 min. After centrifugation, the supernatant was collected and concentrated by rotary evaporation. The residue was redissolved in deionized water and adjusted to a final volume of 1.0 mL as the test solution. An appropriate aliquot of the test solution was used for pre-column derivatization. 100 μL each of derivatization reagent A consisting of triethylamine-acetonitrile solution (1:4, *v*/*v*) and derivatization reagent B consisting of phenyl isothiocyanate-acetonitrile solution (1:80, *v*/*v*) were added, followed by reaction at room temperature for 1 h. The reaction mixture was extracted with n-hexane, and the lower layer was collected, passed through a 0.22 μm microporous filter membrane, and subjected to HPLC analysis. Chromatographic separation was performed on an Acclaim^TM^ 120 C18 column (4.6 mm × 250 mm, 5 μm) at a column temperature of 40 °C. The mobile phases consisted of ammonium acetate buffer (pH 6.5) as phase A and acetonitrile as phase B, delivered at a flow rate of 1.0 mL/min. The injection volume was 5 μL, and detection was carried out at 254 nm.

### 2.9. Determination of Free Sulfhydryl and Reduced Glutathione (GSH) Content

#### 2.9.1. Determination of Free Sulfhydryl Content

Free sulfhydryl content was determined according to the instructions of a commercial free sulfhydryl assay kit (Leda Qibo Biotechnology Co., Ltd., Quanzhou, China). The principle of the assay is as follows: 5,5′-dithiobis-(2-nitrobenzoic acid) (DTNB) reacts with free sulfhydryl groups (-SH) in the sample to produce yellow 2-nitro-5-mercaptobenzoic acid (TNB), which exhibits a characteristic absorption peak at 412 nm. Absorbance is proportional to free sulfhydryl content. Dough extract was reacted with the DTNB working solution, followed by absorbance measurement at 412 nm. Free sulfhydryl content was calculated based on a standard curve and expressed as μmol per gram fresh weight (μmol/g FW).

#### 2.9.2. Determination of GSH Content

Reduced glutathione (GSH) content was determined according to the instructions of a commercial GSH assay kit (Leda Qibo Biotechnology Co., Ltd.). The principle of the assay is as follows: GSH reacts with DTNB to produce TNB and oxidized glutathione (GSSG). Subsequently, GSSG is reduced back to GSH by NADPH under the catalysis of glutathione reductase (GR), thereby establishing a cyclic reaction. This process leads to the continuous generation of TNB, and the rate of increase in absorbance at 412 nm is proportional to the GSH content in the sample. Sample extract was mixed with the reaction working solution containing DTNB, GR, NADPH, and other reagents. The change in absorbance at 412 nm was monitored immediately, and the reaction rate was determined. GSH content was calculated based on a standard curve and expressed as micromoles per gram fresh weight (μmol/g FW).

### 2.10. Size-Exclusion High-Performance Liquid Chromatography (SE-HPLC)

The molecular weight distribution of proteins was determined according to the method described by Yu et al. [24]. Dough (3 mg) was extracted by vortexing with 3 mL of 2% (*w*/*v*) SDS in phosphate-buffered saline (SDS-PBS, 0.05 M, pH 6.8) for 5 h. After centrifugation at 10,000× *g* for 5 min at 4 °C, the supernatant was collected and filtered through a 0.22 μm membrane for SE-HPLC analysis of protein molecular weight distribution. The chromatographic conditions were as follows: TSKgel G3000SWXL column (Tosoh Corporation, Tokyo, Japan) (7.8 × 300 mm), column temperature of 30 °C, UV detection at 214 nm, mobile phase consisting of 0.2% (*w*/*v*) SDS in phosphate-buffered saline (PBS, 0.05 M, pH 6.8), flow rate of 0.7 mL/min, and injection volume of 20 μL. SDS-soluble polymeric (SDS-P), monomeric (SDS-M), and insoluble (SDS-I) fractions were calculated using the following formulas:

SDS-P (%) = AP/AR × 100(3)

SDS-M (%) = AM/AR × 100(4)

SDS-I (%) = 100 − [SDS-P (%) + SDS-M (%)](5)

where AP and AM are the peak areas of SDS-P and SDS-M, respectively, and AR is the peak area of fully reduced proteins. Reduced proteins were extracted with the SDS–PBS buffer described above containing 1% (*w*/*v*) dithiothreitol.

### 2.11. Statistical Analysis

Data are reported as mean ± standard deviation. One-way analysis of variance followed by Duncan’s multiple-range test was performed in IBM SPSS Statistics 26.0, with *p* < 0.05 considered significant. Microsoft Excel 2019 was used for data processing and Origin 2022 for plotting.

## 3. Results and Discussion

### 3.1. Effect of Sourdough Addition on the Rheological Properties of Mixed Fermentation Dough

Table 3 shows that sourdough addition altered dough fermentation (Hm, Tx, Vt, and Rc). Hm increased from 40.50 mm at 0% sourdough to 70.50 mm at 100%. Tx was 49.50 min in the control and increased mainly at ≥50% sourdough, reaching 70.25 min at 100%. Vt peaked at 1766.50 mL with 30% sourdough and fell to 1147.50 mL at 100%, whereas Rc rose from 71.15% to 79.65%. The increase in Hm observed in sourdough-containing doughs suggests enhanced yeast fermentation activity and/or improved dough extensibility under the acidic conditions generated by sourdough fermentation, although the relative contribution of each factor warrants further investigation. However, as previously reported, excessive acidification can depolymerise high-molecular-weight glutenins and weaken the gluten network [25]. This interpretation is consistent with reports that sourdough can improve gas retention and dough elasticity within an appropriate range [26]. The high Vt at 30% suggests that yeast remained active while LAB contributed to co-fermentation [27]. By contrast, the lower Vt at 100% may reflect nutrient competition or acid inhibition of yeast. The high Rc at 100% should be interpreted cautiously because total gas production was low, reducing the amount available for loss. Because industrial proofing is completed before Tx, moderate sourdough addition may increase proofing height without promoting premature gas leakage.

### 3.2. Effect of Sourdough Addition on the Macroscopic Quality of Frozen Raw Steamed Stuffed Buns

#### 3.2.1. Whiteness

**Figure 1:**
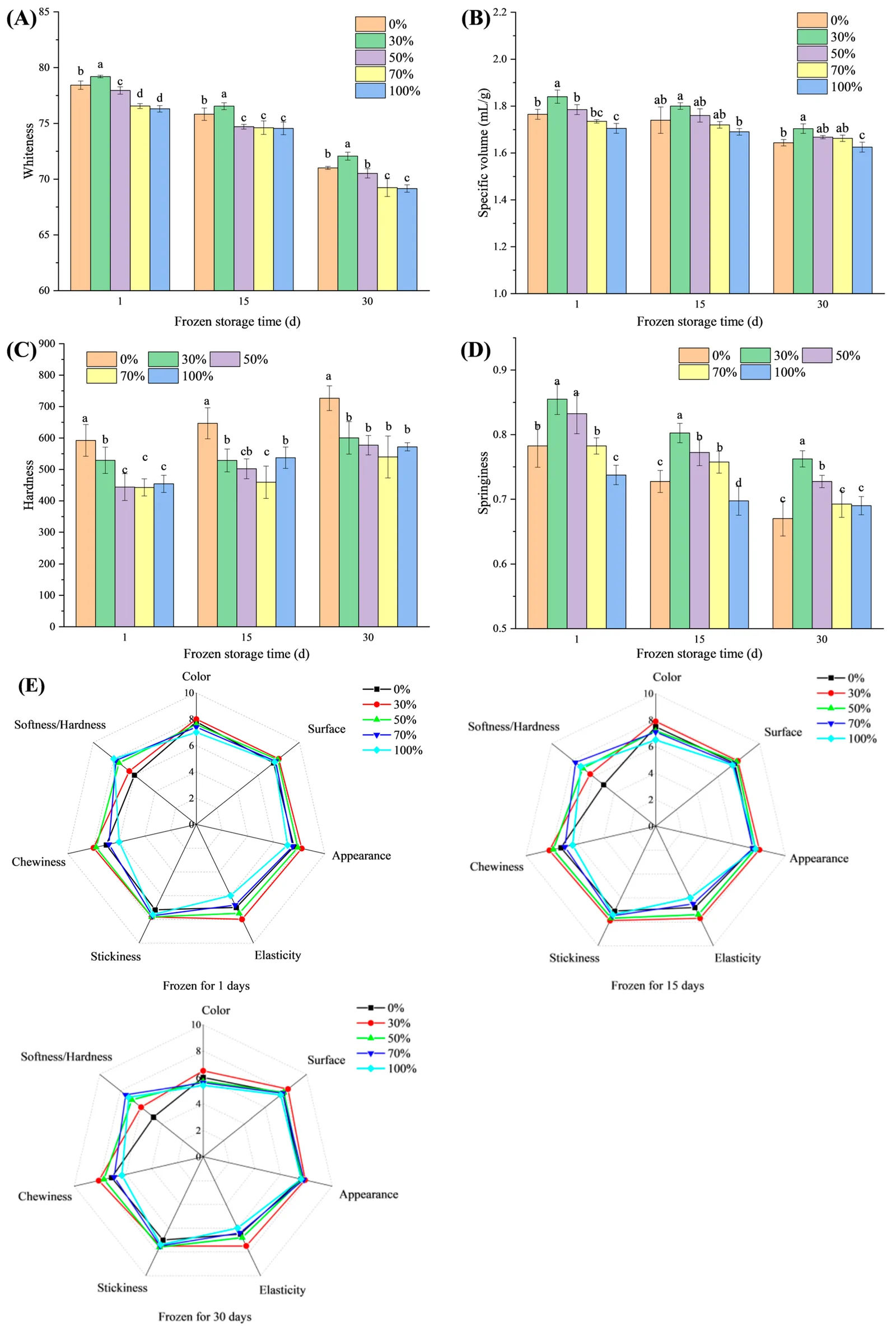
Effects of different sourdough addition levels on the macro-quality of frozen raw steamed stuffed buns: (**A**) whiteness, (**B**) specific volume, (**C**) hardness, (**D**) springiness, and (**E**) sensory evaluation scores. Note: Different lowercase letters indicate significant differences among samples for the same index, and the same convention applies to the figures below. Data were expressed as mean ± standard deviation. Means within the same column followed by different superscript lowercase letters represent significant difference (*p* < 0.05).

The sourdough addition level had a significant effect on the apparent whiteness of frozen raw steamed stuffed buns during 30 days of storage at −18 °C (Figure 1A). On the first day of storage, the whiteness of the frozen raw steamed stuffed buns initially increased and then decreased with increasing sourdough addition. The 30% sourdough group exhibited the highest whiteness value, whereas the 0% sourdough group (control) and 100% sourdough group showed relatively lower whiteness. As frozen storage progressed to 15 and 30 days, the whiteness of all experimental groups continued to decline. At the end of the storage period (30 days), significant differences in whiteness among the groups remained. The 30% sourdough group maintained the highest whiteness, which was significantly greater than that of other groups. In contrast, groups with sourdough addition levels exceeding 50% showed a more rapid decline in whiteness, with final values significantly lower than that of the control group. On day 1, appropriate sourdough addition, particularly at 30%, significantly improved the whiteness of the frozen raw steamed stuffed buns. This improvement was mainly attributed to the optimized dough microstructure induced by sourdough. Sourdough fermentation promotes the formation of a finer and more uniform pore structure, thereby enhancing the overall color and brightness of the frozen raw steamed stuffed buns. However, when the sourdough addition level was excessive, such as 100%, the dough became stickier due to prolonged fermentation. This reduced the surface smoothness of the prepared buns, directly impairing their light reflection properties and reducing whiteness. As frozen storage time increased, the whiteness of all samples generally decreased. This decline was mainly attributed to complex biochemical reactions occurring during frozen storage. This decrease may be attributed to the disruption of surface microstructure caused by ice crystal formation and recrystallization during frozen storage, accompanied by moisture redistribution and surface dehydration that increase light scattering, thereby reducing the apparent whiteness. The results indicate that, throughout the entire frozen storage period, 30% sourdough addition was the most effective in maintaining the whiteness stability of frozen raw steamed stuffed buns. The observed whiteness differences were also accompanied by significant variations in sensory color scores, suggesting that such differences are perceivable in practical consumption contexts. This effect may be related to the balance achieved at this addition level among the gluten network structure, moisture distribution, and microbial metabolic activity, thereby delaying colour deterioration.

#### 3.2.2. Specific Volume

Specific volume is a key indicator for evaluating dough quality because it directly reflects the degree of volume expansion and gas-retention capacity of flour-based products [28]. As shown in Figure 1B, under frozen storage at −18 °C, different sourdough addition levels (0%, 30%, 50%, 70%, and 100%) had a significant effect on the specific volume of frozen raw steamed stuffed buns. On the first day of storage, the specific volume initially increased and then decreased with increasing sourdough addition, with the 30% sourdough group exhibiting a significantly higher value than the other groups. As storage time increased to 15 and 30 days, the specific volume of all experimental groups continuously decreased. Throughout the entire storage period, the specific volume of the 30% sourdough group remained the highest and showed the most gradual decline. In contrast, the 70% and 100% sourdough groups showed no clear advantage in specific volume even at the initial stage of storage, and their deterioration became more pronounced during subsequent frozen storage. These results indicate that the effect of sourdough addition on the specific volume of frozen raw steamed stuffed buns was not linearly dose-dependent. Although fermentation rheological analysis suggested that increasing sourdough addition improved the maximum dough height (Hm) during fermentation, excessive sourdough addition, such as 70% and 100%, had an adverse effect on maintaining the specific volume of frozen raw steamed stuffed buns after freezing and prolonged frozen storage. Moderate weakening of the gluten network can reduce its resistance to CO_2_ expansion during fermentation, thereby facilitating gas expansion and increasing specific volume [29]. However, excessive sourdough introduces excessive acidic substances, resulting in overly high dough acidity, which severely disrupts the continuity of the gluten network. This weakens its ability to encapsulate and retain fermentation gases and ultimately reduces product specific volume [30]. The general decline in specific volume across all samples during frozen storage was mainly attributed to changes in water status and ice crystal damage within the system. Prolonged storage promotes the conversion of part of the bound water into freezable water. The greater number of ice crystals and potential recrystallization cause continuous physical damage to the formed gluten network, weakening the gas-holding capacity of the dough [31]. Concurrently, the frozen environment also reduces yeast activity and gas production capacity [32]. Together, these factors lead to reduced gas retention and volume shrinkage. In this study, 30% sourdough effectively mitigated the decline in specific volume induced by frozen storage. This suggests that, at this addition level, the complex microbial community and its metabolites in sourdough contributed to the formation of a more stable and resilient gluten network structure. This network exhibited stronger resistance to damage under frozen storage stress, thereby better maintaining the gas-retention capacity and volume stability of frozen raw steamed stuffed buns. Overall, the 30% sourdough addition level exhibited the best quality improvement in this study. However, its potential limitations should also be noted. The activity of the starter culture, wheat flour characteristics, and activation conditions can all influence sourdough preparation, and its industrial application therefore requires further validation.

#### 3.2.3. Textural Properties

The textural properties of frozen raw steamed stuffed buns, particularly hardness and springiness, are key determinants of mouthfeel and consumer acceptance. Figure 1C,D clearly demonstrate the significant effects of sourdough addition level and frozen storage time on these two indicators. For hardness (Figure 1C), on the first day of frozen storage, the hardness of the frozen raw steamed stuffed buns gradually decreased with increasing sourdough addition. The 0% sourdough group (control) exhibited the highest hardness, whereas the 100% sourdough group showed the lowest value. As the frozen storage time increased to 15 and 30 days, the hardness of all experimental groups displayed a consistent increasing trend. Throughout the entire frozen storage period, the control group maintained the highest hardness and showed the greatest increase. In contrast, the sourdough-added groups exhibited significantly lower hardness values and smaller increases than the control group, with the 30% to 50% sourdough addition range showing a more balanced inhibitory effect. For springiness (Figure 1D), on the first day of frozen storage, the springiness of frozen raw steamed stuffed buns initially increased and then decreased with increasing sourdough addition, with the 30% sourdough group demonstrating significantly higher springiness than all other groups. As frozen storage time increased, the springiness of all samples decreased. Throughout storage, the 30% sourdough group maintained the highest springiness and exhibited the best stability. The control group showed the most pronounced decrease in springiness. Although the high-addition groups (70% and 100%) showed better springiness than the control group, their values remained lower than those of the 30% sourdough group. Changes in the hardness of frozen raw steamed stuffed buns are primarily attributed to ice-crystal-induced damage and alterations in the gluten network during frozen storage. At the initial stage of frozen storage, sourdough addition effectively reduced product hardness. This effect was attributed to the organic acids and metabolites produced during sourdough fermentation, which softened the gluten network structure. However, during prolonged frozen storage, the continuous formation and growth of ice crystals caused persistent mechanical damage to the gluten network. Concurrently, the decline in yeast viability and the reduction in gas-retention capacity collectively contributed to the increased hardness [5]. The slower increase in hardness observed in the sourdough-added groups may be attributed to microbial metabolites present in sourdough, such as low-molecular-weight peptides, sugars, and certain enzymes. These components may help protect or stabilise the gluten network, mitigating physical damage caused by ice crystals and thereby slowing the hardening process to some extent. Changes in springiness were regulated in a more complex manner by the diverse enzymatic activities present in sourdough. The improved springiness observed at an appropriate sourdough addition level, particularly 30%, may be associated with moderate enzyme-mediated modifications of proteins and starch, which could influence the extensibility and recoverability of the gluten network [33]. At higher sourdough addition levels, more extensive structural modification of gluten proteins and starch may have contributed to the reduced springiness [34]. However, because protease and amylase activities were not directly measured in this study, these mechanisms remain hypothetical and require further experimental verification. Overall, 30% sourdough most effectively optimized and maintained the textural properties of frozen raw steamed stuffed buns. It showed advantages in reducing initial hardness, delaying hardening during frozen storage, and achieving optimal springiness. This balance may reflect moderate network modification together with reduced freeze-induced damage.

#### 3.2.4. Sensory Evaluation

Sensory quality declined in all groups during frozen storage (Figure 1E). On day 1, the 30% group received the highest scores for surface gloss, pore fineness, and springiness. The control was markedly sticky, whereas the 100% group scored poorly for shrinkage, springiness, and chewiness. The sensory trends agreed with specific-volume and texture measurements. Appropriate sourdough may improve pore uniformity, water retention, flavour, and extensibility through organic acids and exopolysaccharides. During storage, moisture migration and ice-crystal growth disrupted the crumb structure and reduced gloss, springiness, and chewiness. The 30% and 50% groups showed smaller sensory losses, suggesting better stability of the gluten–starch–water matrix. Overall, 30% sourdough provided the best sensory balance across storage.

### 3.3. Effects of Sourdough Starter Addition Levels on Microbial Metabolism in Mixed Fermentation Dough Systems

#### 3.3.1. pH and TTA

**Figure 2:**
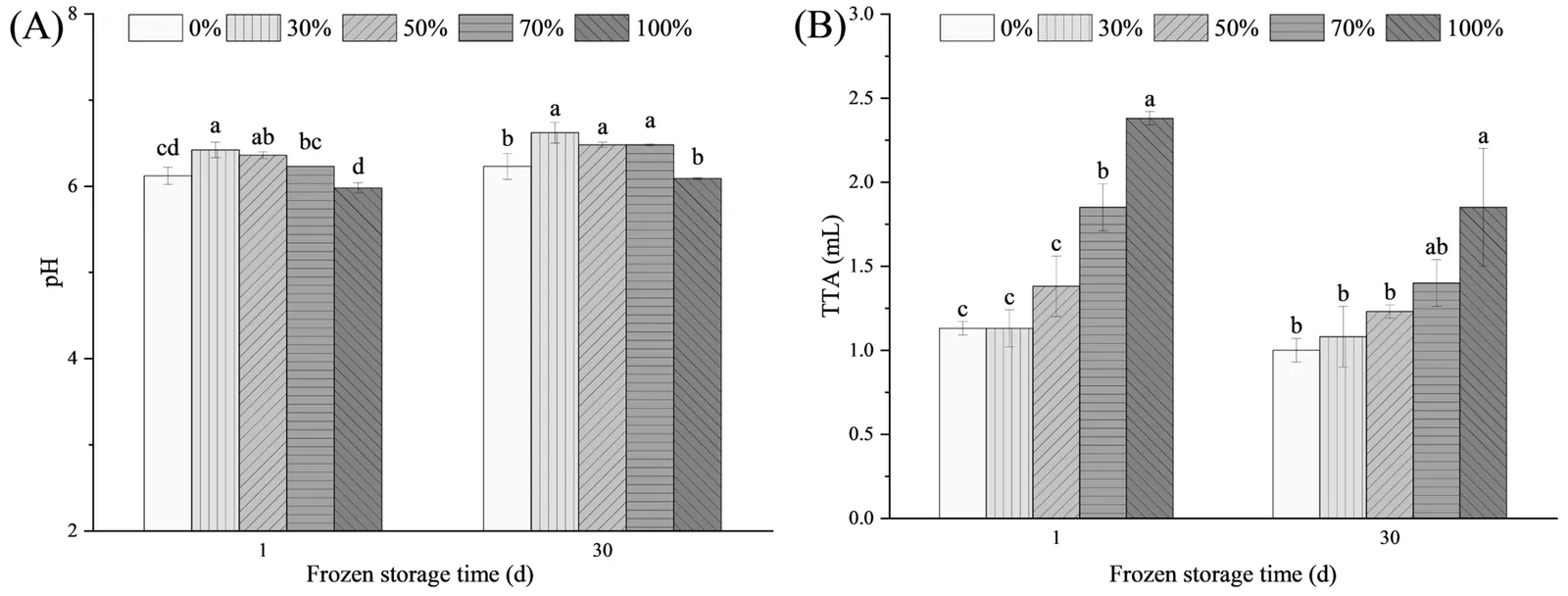
Effects of different sourdough addition levels on dough pH and TTA during frozen storage: (**A**) pH; (**B**) TTA. Note: Data were expressed as mean ± standard deviation. Means within the same column followed by different superscript lowercase letters represent significant difference (*p* < 0.05).

Dough pH and TTA changed with sourdough level and frozen storage (Figure 2). On day 1, pH peaked at 6.42 with 30% sourdough and fell to 5.98 at 100%. TTA increased with sourdough addition and reached 2.38 mL at 100%. After 30 days, pH increased and TTA decreased in all groups, although TTA remained highest at 100% (1.85 mL). LAB and yeasts produce lactic and acetic acids from carbohydrates, so the dose-dependent increase in TTA reflects greater acid accumulation [33]. We hypothesize that the opposing changes in pH and TTA during storage may reflect reduced microbial activity and changes in acid distribution or buffering capacity, although this interpretation requires direct experimental verification. The present measurements do not distinguish among these processes. Nevertheless, the results show that sourdough level controls acidification and may thereby influence microbial activity, gluten stability, and product quality.

#### 3.3.2. LAB and Yeast Content

**Figure 3:**
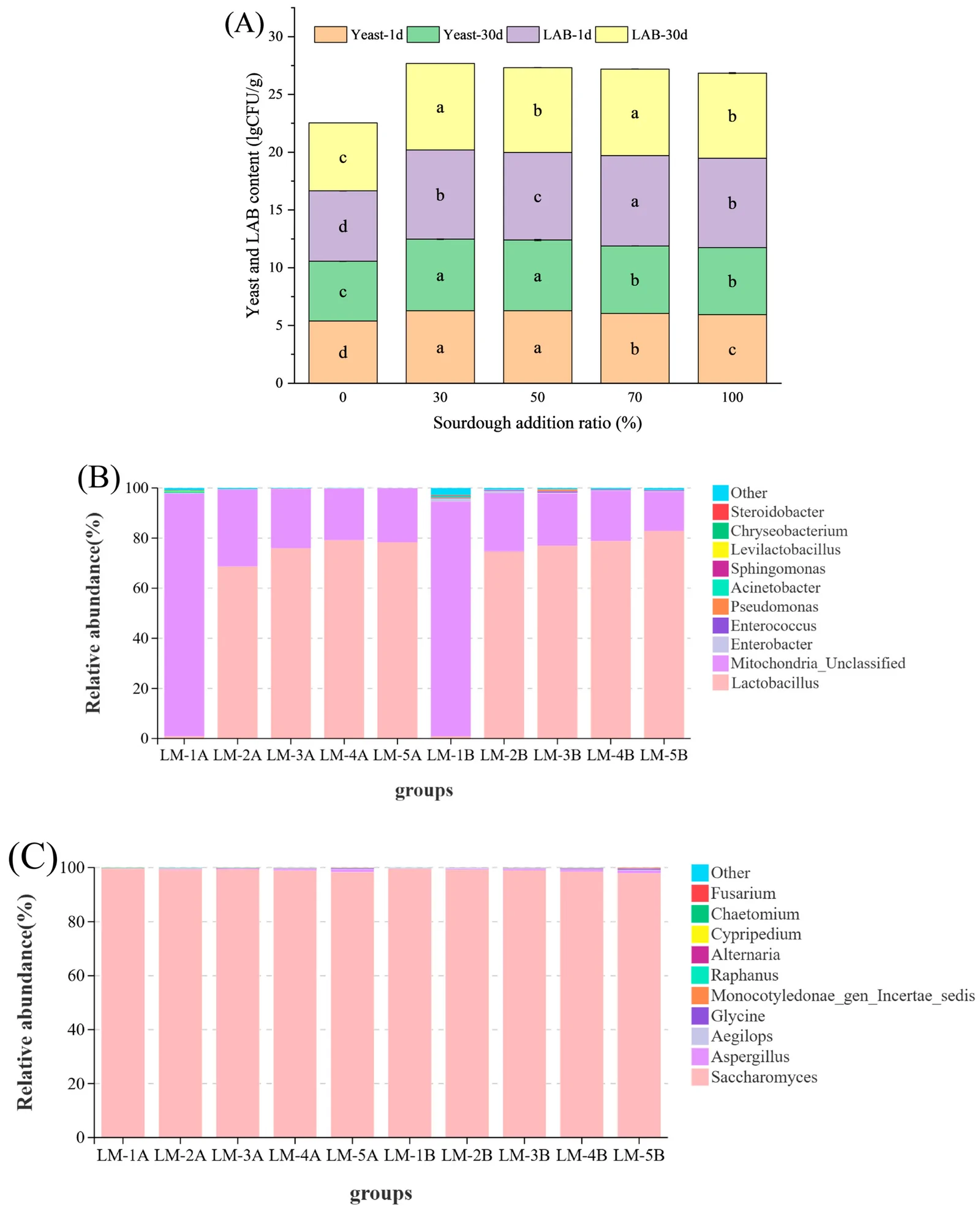
Effects of different sourdough addition levels on the microbial community of dough during frozen storage: (**A**) LAB and yeast content; (**B**) bacterial community composition at the genus level (16S rRNA high-throughput sequencing); (**C**) fungal community composition at the genus level (ITS high-throughput sequencing). Note: Data were expressed as mean ± standard deviation. Means within the same column followed by different superscript lowercase letters represent significant difference (*p* < 0.05). In panels (**B**) and (**C**), LM-A and LM-B represent sample storage for 1 day and 30 days, respectively.

As shown in Figure 3A, the sourdough addition level significantly affected the counts of yeasts and LAB in the frozen dough. On day 1 of frozen storage, the yeast count initially increased and then decreased with increasing sourdough addition, reaching its highest level in the 30% and 50% sourdough groups (6.27 lg CFU/g), which was significantly higher than that in the control group (5.39 lg CFU/g). However, when the addition level increased to 70% and 100%, the yeast count began to decline. In contrast, the LAB count continued to increase significantly with increasing sourdough addition, reaching a maximum in the 70% sourdough group (7.82 lg CFU/g). After 30 days of frozen storage, microbial counts decreased in all groups. Nevertheless, the 30% and 50% sourdough groups still maintained relatively high yeast and LAB counts, demonstrating better survival rates. High-throughput sequencing analysis of the microbial community structure (Figure 3B,C) further revealed compositional changes. At the genus level, *Saccharomyces* was absolutely dominant in all samples. On day 1 of frozen storage, its relative abundance showed little variation among the different addition groups, ranging from 96.79% to 97.92%. After 30 days of storage, the abundance of this genus generally decreased, and the extent of reduction was positively correlated with the sourdough addition level, with the 100% sourdough group showing the greatest decrease (2.21%). Conversely, the relative abundance of *Lactobacillus* increased significantly with increasing sourdough addition on day 1, reaching as high as 66.27% in the 70% sourdough group. After 30 days of frozen storage, its abundance increased rather than decreased in the 30% and 100% sourdough groups. In a co-fermentation system, yeast and LAB exhibited a complex interactive relationship, forming a mutualistic consortium that influenced their growth and metabolism [35]. The results of this study indicate that 30–50% sourdough was associated with higher yeast counts. On the one hand, the sourdough itself introduces initial populations of both yeast and LAB. On the other hand, at this proportion, the two types of microorganisms may have established a favorable symbiotic relationship. As previously documented [36], yeast provides LAB with growth factors such as pyruvate, vitamins, and amino acids, thereby promoting their proliferation. In turn, amino acids and other metabolites produced by LAB through proteolysis can reciprocally support yeast growth [37]. However, when excessive sourdough (≥70%) was added, yeast growth was inhibited. This was likely due to the high proportion of LAB competing with yeast for limited nutrients, while the substantial accumulation of acidic metabolites altered the microenvironment and suppressed yeast activity [9]. During frozen storage, microorganisms are exposed to low-temperature stress. The results demonstrate that a moderate sourdough addition (30% to 50%) helped maintain the viability and population stability of both yeast and LAB throughout frozen storage. This may be attributed to the activation of stress response mechanisms in yeast cells under these conditions, leading to the accumulation of more protective metabolites and consequently enhanced freezing tolerance [38]. Moreover, low-temperature adaptation of the starter may have conferred enhanced cold tolerance, particularly among its LAB strains, which helps maintain a relatively stable community structure and numerical dominance during frozen storage. In summary, the sourdough addition level directly influenced the dynamics and frozen storage stability of the microbial community by modulating the ratio and interaction between yeast and LAB within the fermentation system. These changes can exert a substantial impact on the fermentation characteristics and final quality of the product. There was a clear correlation trend between the microbial count results and the dough quality parameters. After 30 days of frozen storage, treatments with higher viable counts of yeast and lactic acid bacteria (especially the 30% sourdough addition group) exhibited higher specific volume. Meanwhile, the maintenance of yeast viability was positively correlated with the reduction in product hardness and the increase in springiness. The 30% addition group showed the smallest increase in hardness and the best retention of springiness after 30 days of frozen storage, whereas the control group exhibited a significant increase in hardness and a marked decrease in springiness. Previous studies have also shown that the microbial community composition of sourdough plays a key role in influencing dough properties and ultimately affects product quality [39].

#### 3.3.3. Metabolic Accumulation of Organic Acids and Ethanol

The accumulation of key metabolites in dough with different sourdough addition levels during frozen storage is presented in Table 4. Regarding organic acids, lactic acid content increased significantly with increasing sourdough addition and continued to accumulate throughout frozen storage. On day 1 of frozen storage, the lactic acid content in the 100% sourdough addition group (4.36 mg/g) far exceeded that of the control group (0.03 mg/g). After 30 days of frozen storage, lactic acid content further increased in all groups, reaching 6.57 mg/g in the 100% sourdough group. Acetic acid content showed a slight increase with increasing sourdough addition on day 1; however, after 30 days of storage, differences among groups narrowed, and a decrease was observed in some groups. Ethanol content showed a different trend, reaching its highest level at 50% sourdough addition (1.14 mg/g) on day 1, which was higher than that of the control group (0.88 mg/g). After 30 days of frozen storage, ethanol content generally decreased, although the 70% sourdough group maintained the highest level (0.77 mg/g). In addition, EPS content increased progressively with increasing sourdough addition, with the 100% sourdough group showing the highest EPS content (10.0 g/kg), which was significantly higher than that of the control group (8.2 g/kg). The production of organic acids is a direct indicator of LAB metabolic activity and plays a key role in shaping the flavor and texture of the final product. The accumulation of lactic acid and acetic acid is the primary cause of dough acidification. Lactic acid contributes to a milder sourness and helps promote the formation of more elastic dough, whereas acetic acid contributes to a firmer structure [40]. In this study, the lactic acid content was significantly higher than that of acetic acid. This was directly related to the substantial increase in lactic acid production driven by dominant bacterial communities such as *L. sanfranciscensis*, which efficiently utilize maltose for acid production [41]. The increase in organic acid content during frozen storage may be attributed to two factors. On the one hand, lactic acid bacteria may maintain a low level of metabolic activity under frozen conditions. The relative contribution of each mechanism requires further experimental differentiation. On the other hand, ice crystal formation during frozen storage leads to an increase in the effective concentration of solutes (including organic acids) in the unfrozen water phase, known as the cryoconcentration effect. This activity may further activate proteases, thereby affecting the gluten network [20]. Ethanol is primarily produced through the fermentative metabolism of fermentable sugars by yeast. Higher early ethanol at moderate sourdough reflected active yeast fermentation. However, its decline after long-term frozen storage may be related to decreased yeast viability. Notably, the relatively higher ethanol content observed after frozen storage at specific addition levels, such as 70%, may be associated with particular interaction patterns between yeast and LAB. Previous studies have indicated that *L. sanfranciscensis* can inhibit the production of flavor compounds such as ethanol by *S. cerevisiae* [42]. During frozen storage, yeast continues to ferment and produce metabolites such as ethanol and CO_2_, which negatively impact the gluten network and specific volume of the dough [43]. As previously established [44,45,46,47,48], during freezing and subsequent frozen storage, yeast cells trigger a range of stress defense mechanisms, including the oxidative stress response, heat shock response, and the HOG pathway, leading to the accumulation of protective metabolites such as trehalose, glycerol, amino acids, glutathione, and heat shock proteins, which enhance stress tolerance. Specifically, ethanol interacts with wheat gliadin, impairing its ability to stabilize gas cells [49], while CO_2_ accelerates the destabilization of the frozen dough system [50].

#### 3.3.4. Fermentable Sugar Content

As shown in Table 5, sourdough addition significantly altered the composition and dynamics of fermentable sugars in frozen dough. On day 1 of frozen storage, fructose was the most abundant sugar in the control group without sourdough (0%), with a content of 13.14 mg/g. With sourdough addition, the sugar composition changed markedly: maltose became the predominant sugar, and its content increased significantly as the sourdough addition level increased from 0% to 100% (from 9.33 mg/g to 28.28 mg/g). Concurrently, sucrose content also increased notably with sourdough addition, whereas fructose content decreased. Total fermentable sugar content increased significantly with increasing sourdough addition. After 30 days of frozen storage, the total sugar content in all experimental groups remained stable or even increased slightly. The slight increase in total fermentable sugars during frozen storage may be attributed to the following factors. First, ice crystal formation during frozen storage causes physical damage to starch granules, increasing the content of damaged starch and thereby enhancing the accessibility of amylases to their substrates. Second, although enzymatic activities are significantly reduced at −18 °C, their residual activity can still act over prolonged storage, and the cryoconcentration effect may partially sustain local reaction efficiency. These interpretations remain speculative and warrant further experimental verification. Glucose content generally showed slight accumulation, whereas fructose content exhibited a widespread decline. The observed changes in fermentable sugar composition reveal the profound impact of the microbial enzyme system derived from sourdough on the dough matrix. After sourdough addition, maltose became the dominant sugar, indicating that the rate of starch hydrolysis by amylase during fermentation exceeded the rate of maltose utilization by microorganisms [51]. Concurrently, sucrose accumulation may be attributed to the activation of endogenous starch amylase by sourdough, thereby accelerating starch degradation into glucose [52]. As a result, yeast preferentially consumed glucose, which slowed down sucrose decomposition. In the later stages of frozen storage, glucose accumulation and fructose decline reflected the combined effects of slow amylase-mediated hydrolysis and limited fructose utilization by microorganisms under low-temperature inhibition. The effects of different sourdough addition levels on sugar metabolism showed clear distinctions: high addition levels (70–100%) promoted sugar accumulation during frozen storage, whereas moderate addition levels (30–50%) likely achieved a better balance between metabolic activity and stability, suggesting a different balance between sugar release and microbial utilisation.

#### 3.3.5. Free Amino Acid Content

**Figure 4:**
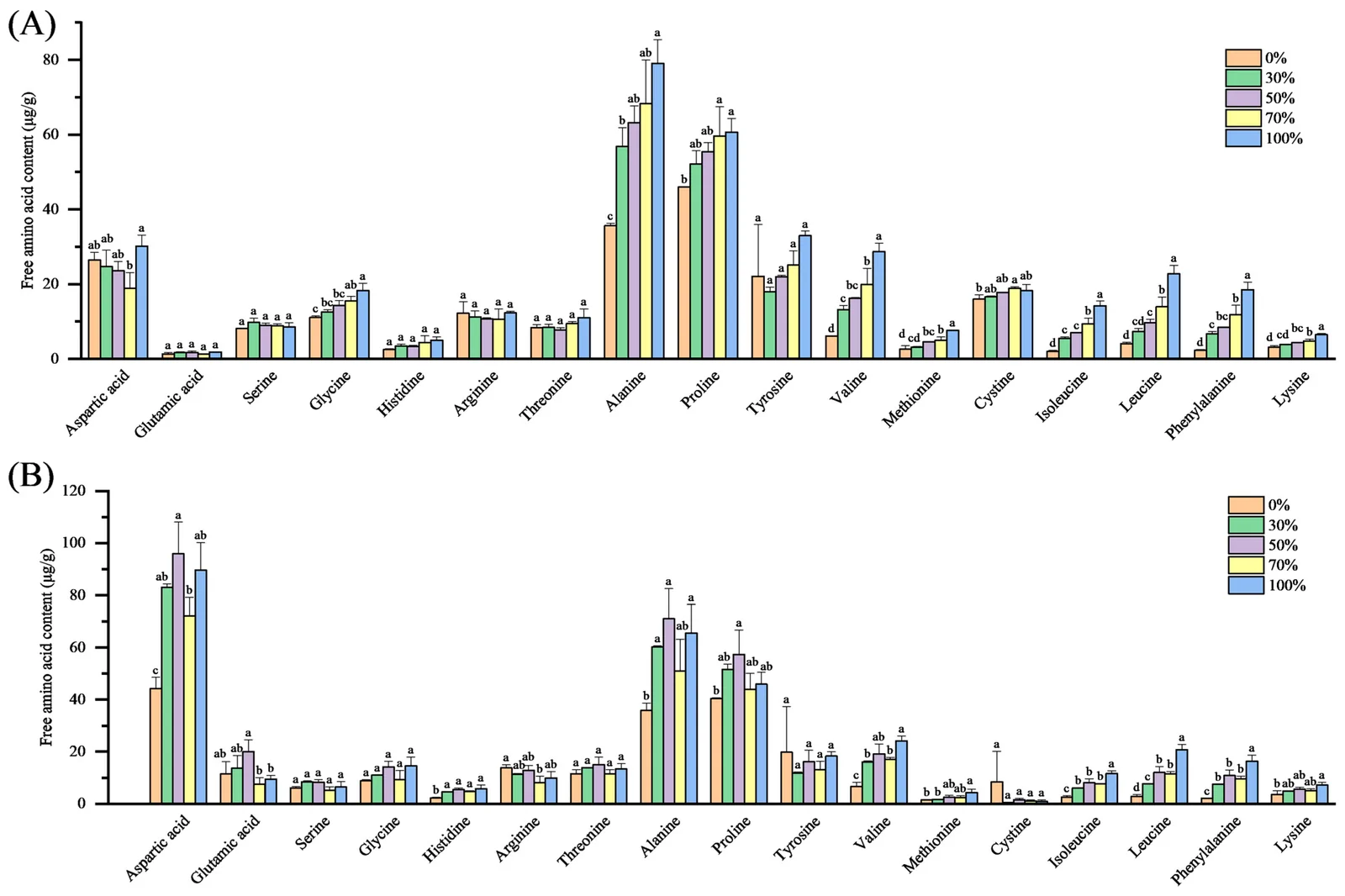
Effect of different sourdough addition levels on the free amino acid content in dough during frozen storage. Note: Data were expressed as mean ± standard deviation. Means within the same column followed by different superscript lowercase letters represent significant difference (*p* < 0.05). Figure (**A**) and Figure (**B**) represent sample storage for 1 day and 30 days, respectively.

The compositional changes in free amino acids are shown in Figure 4. On day 1 of frozen storage, the total free amino acid content in the dough increased significantly with increasing sourdough addition. Specifically, the concentrations of various amino acids, including glycine, alanine, proline, valine, methionine, isoleucine, leucine, phenylalanine, and lysine, increased significantly with higher sourdough addition levels. After 30 days of frozen storage, changes in amino acid composition varied among the different addition groups: the contents of aspartic acid and glutamic acid increased significantly, whereas the contents of methionine and cystine decreased notably. Within the range of 0% to 50% sourdough addition, the total free amino acid content increased. However, at the higher addition levels of 70% and 100%, the total content declined. This non-linear response suggests that excessive sourdough addition may alter proteolytic dynamics or microbial nitrogen metabolism under extended frozen storage conditions. The significant increase in free amino acid content was mainly attributed to the acidic environment generated by sourdough fermentation, which promoted flour-protein hydrolysis and the subsequent release of amino acids [37]. Concurrently, peptidases secreted by LAB further degraded polypeptides into amino acids. Previous studies have indicated that sourdough fermentation significantly increases the concentration of free amino acids, particularly glutamic acid, proline, and aspartic acid. Among these amino acids, proline accumulation may reflect the proteolytic activity of the sourdough microbiota. Furthermore, as a known cryoprotectant, proline accumulation contributes to enhancing yeast freeze tolerance. It has been reported [53] that the intracellular proline content of yeast cells in frozen dough is a key factor affecting their freeze tolerance. Proline accumulation endows yeast with enhanced freeze–thaw resistance and enables them to maintain higher fermentation capacity after freezing. The changes in amino acid composition after frozen storage, particularly the increase in aspartic acid and glutamic acid and the decrease in methionine, were related to metabolic regulation under stress conditions. These shifts may reflect stress-dependent microbial nitrogen metabolism, although gene expression was not measured here. Glutamic acid, proline, and lysine are key amino acids affecting the elasticity of gluten proteins. Notably, the 30% and 50% addition groups exhibited both relatively higher levels of these amino acids and superior product elasticity. However, whether the former contributes to the latter remains to be established. These changes collectively demonstrate that sourdough addition optimizes dough quality in flavour and functional properties by regulating proteolysis and the metabolism of specific amino acids.

#### 3.3.6. Free Sulfhydryl Group Content

**Figure 5:**
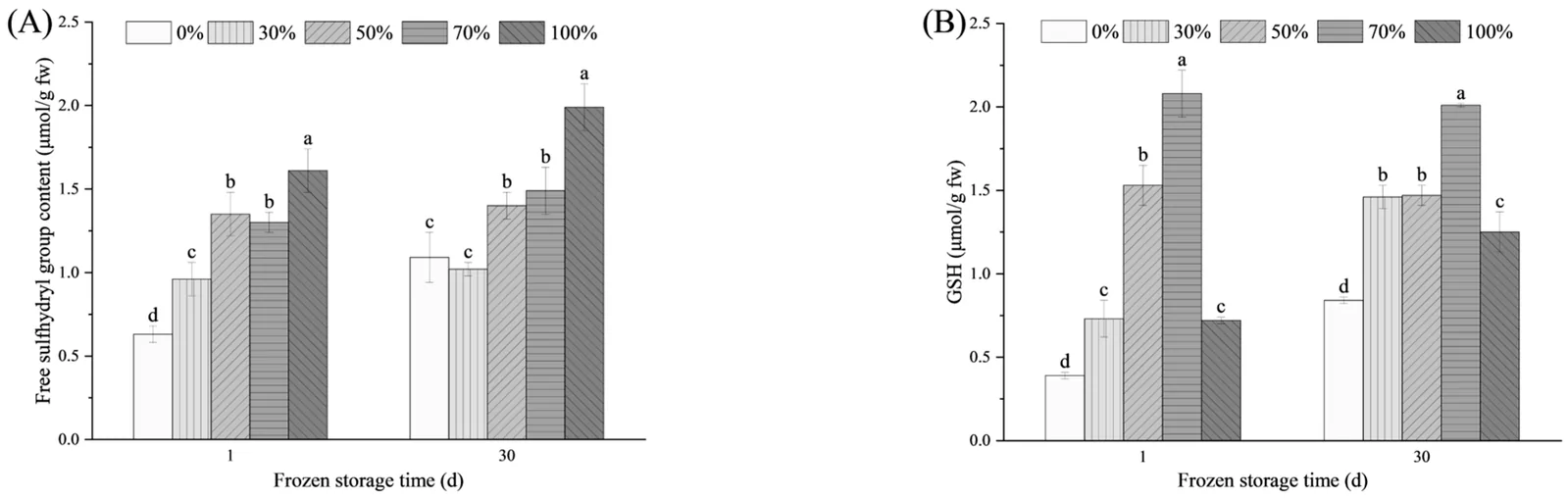
Effect of different sourdough addition levels on the content of free sulfhydryl groups and glutathione in dough during frozen storage: (**A**) free sulfhydryl group content; (**B**) GSH content. Note: Data were expressed as mean ± standard deviation. Means within the same column followed by different superscript lowercase letters represent significant difference (*p* < 0.05).

The changes in free sulfhydryl content in frozen dough with different sourdough addition levels during storage are shown in Figure 5A. At the initial stage of frozen storage (day 1), the free sulfhydryl group content in the dough increased significantly with increasing sourdough addition. The control group without sourdough (0%) showed the lowest content (0.63 µmol/g fresh weight), while the 100% sourdough group exhibited the highest content (1.61 µmol/g fresh weight). After 30 days of frozen storage, the free sulfhydryl group content increased in all groups, although the extent of increase varied significantly. The control group showed the largest increase, reaching 73.02%, whereas the 30% and 50% sourdough groups exhibited minimal increases of only 6.25% and 3.70%, respectively, indicating that their free sulfhydryl group content remained relatively stable during storage. Nevertheless, the 100% sourdough group still showed the highest free sulfhydryl group content (1.99 µmol/g fresh weight) among all groups after 30 days of frozen storage. The content of free sulfhydryl groups (-SH) is a key indicator for characterizing the dynamic balance of disulfide bonds (-S-S-) and the structural stability of gluten proteins. The gluten network is mainly composed of glutenin, which forms intermolecular disulfide cross-links, and gliadin, which relies on intramolecular disulfide bonds and other interactions. In this study, the substantial increase in free sulfhydryl group content observed in the control group after frozen storage indicated that the disulfide bonds maintaining the higher-order structure of gluten proteins were disrupted under frozen-storage stress. The increase in free sulfhydryl content is generally regarded as an indirect indicator of disulfide bond cleavage, since the disruption of disulfide bonds in the gluten network releases free thiol groups. However, it should be noted that the present study did not directly determine the disulfide bond content or directly characterize the cleavage process; therefore, the increase in free sulfhydryl content serves only as indirect evidence of disulfide bond disruption, rather than direct proof. This disruption generated more free sulfhydryl group, leading to protein depolymerization and weakening of the network structure, thereby impairing the processing properties of the dough and the quality of the final product. After sourdough addition, the initial free sulfhydryl group content in the dough was significantly higher than that in the control group. However, the key change was observed during frozen storage: appropriate sourdough addition (30% to 50%) effectively inhibited the excessive increase in free sulfhydryl groups during storage. This indicates that certain components in the sourdough system, such as specific microbial metabolites or antioxidant substances, contributed to stabilizing the protein structure, slowing frozen storage-induced disulfide-bond cleavage, and thereby maintaining the integrity of the gluten network. Although excessive sourdough addition (100%) resulted in the highest initial and final free sulfhydryl group contents, its increase during storage was still lower than that of the control group, also indicating a certain stabilizing effect. However, other adverse changes in the network structure may have occurred due to excessive acidification or proteolysis. Therefore, from the perspective of maintaining protein structural stability during frozen storage, a sourdough addition level of 30% to 50% represents a more suitable range.

#### 3.3.7. GSH Content

GSH content increased and then decreased with sourdough on day 1, peaking at 2.08 μmol/g fresh weight at 70% (Figure 5B). After 30 days, GSH increased in the control, 30%, and 100% groups but remained stable at 50% and 70%. The control showed the largest increase (115.38%), whereas the 70% group retained the highest final level (2.01 μmol/g). The sourdough itself contained 0.65 ± 0.04 μmol/g GSH. Sourdough can introduce GSH and increase microbial biomass, while some LAB may support glutathione cycling [47]. The marked control increase after storage may reflect GSH release from freeze-damaged yeast cells. Stable GSH at 50–70% is therefore consistent with better cell integrity or altered GSH turnover. Nevertheless, GSH can reduce glutenin disulfide bonds and weaken dough. The 70% group did not show the best product quality, indicating that GSH stability alone does not determine the optimal sourdough level.

#### 3.3.8. Protein Molecular Weight Distribution

The results for protein molecular weight distribution (Table 6) show the effects of sourdough addition level and frozen storage time on the structural state of gluten proteins. On day 1 of frozen storage, SDS-insoluble proteins (SDS-I) accounted for 87.87% of the sample without sourdough addition, constituting the predominant fraction of gluten proteins. After the addition of 50% sourdough, SDS-I sharply decreased to 52.72%, whereas the proportions of SDS-soluble polymers (SDS-P) and monomeric proteins (SDS-M) increased significantly to 12.13% and 35.16%, respectively. When the sourdough addition level was increased to 100%, SDS-M further increased substantially to 59.99%, becoming the dominant component. Concurrently, SDS-I decreased to 36.09%, and SDS-P declined to 3.92%. After 30 days of frozen storage, the protein distribution patterns of all samples converged towards a similar state: the proportion of SDS-P decreased sharply to below 0.2% in all groups, the proportion of SDS-I rebounded significantly, and the proportion of SDS-M also changed accordingly. These changes are consistent with the depolymerization and reorganization behavior of gluten proteins during fermentation and frozen storage. At the initial stage of frozen storage, sourdough addition significantly induced protein structural depolymerization. In the absence of sourdough, gluten proteins primarily existed as highly cross-linked large molecular aggregates, as indicated by the high SDS-I proportion, forming a dense network structure. After sourdough addition, particularly at 50%, the decrease in SDS-I accompanied by the simultaneous increase in SDS-P and SDS-M indicated that active components in sourdough, such as reducing substances or proteases produced by microbial metabolism, disrupted the forces maintaining the higher-order structure of proteins, including disulfide bonds. This caused some insoluble large molecular aggregates to depolymerize into soluble polymers and monomers. When the sourdough addition level was excessively high, as in the 100% group, the proportion of SDS-P decreased while SDS-M further increased, suggesting that the proteins underwent continuous and more extensive depolymerization, with small molecular polymers likely being further degraded. After 30 days of frozen storage, the disappearance of SDS-P and the general recovery of SDS-I in all groups indicated significant protein reorganization under long-term frozen conditions. Ice crystals formed during frozen storage can disrupt the original protein structure and expose more hydrophobic groups. Subsequently, these depolymerized protein molecules reassemble through non-covalent forces, such as hydrophobic and electrostatic interactions, forming aggregates with higher molecular weight and lower solubility. This depolymerisation–reorganisation process may contribute to the hardening of the gluten network and the decrease in extensibility induced by frozen storage. Sourdough level may therefore influence the texture of the final product by regulating the balance of this process. Moderate depolymerization, such as that induced by 50% sourdough addition, can enhance network extensibility, imparting a soft and airy texture to the product while retaining a sufficient structural framework to maintain its shape. However, excessive depolymerization, such as that observed with 100% sourdough addition, severely compromises the continuity and supportive capacity of the network, leading to a crumbly structure and sticky mouthfeel. Thus, an intermediate sourdough level may better balance protein extensibility and network continuity.

## 4. Conclusions

Sourdough–yeast co-fermentation improved the frozen-storage quality of raw steamed stuffed buns. Based on the experimental results of this study, a sourdough addition level of 30% is recommended as the optimal choice under the specific formulation and processing conditions tested. It should be noted, however, that the optimal level may vary depending on flour characteristics, sourdough microbiota, or processing conditions. This formulation combined high specific volume, lower hardness, greater springiness, and the highest sensory scores during 30 days at −18 °C. Moderate sourdough addition also maintained comparatively high yeast and LAB counts and altered organic acids, sugars, amino acids, and exopolysaccharides. Free sulfhydryl content changed least at 30–50%, whereas GSH remained most stable at 50–70%; these distinct responses should not be conflated. SE-HPLC further showed that intermediate sourdough limited the excessive protein depolymerisation observed at 100%. Together, these results associate quality retention with balanced microbial activity, metabolite accumulation, and preservation of gluten structure. Because these mechanisms were inferred from parallel measurements, targeted experiments are needed to establish causality. Within the tested formulation and storage conditions, 30% sourdough is therefore the most suitable level for industrial evaluation.

Several limitations of this study should be acknowledged. First, some mechanistic explanations were primarily based on inferences from the literature and correlation analyses, lacking direct support from targeted quantitative experiments. Furthermore, this study was conducted at the laboratory scale, and validation at the industrial scale will be an important step for future translation and application. Future research could be pursued in the following directions: (1) verifying the accumulation and function of cryoprotective substances through targeted metabolomics; (2) extending the frozen storage period to evaluate long-term quality stability; (3) exploring the optimal addition levels under different combinations of flour and sourdough microbiota; and (4) validating the process feasibility at pilot or industrial scale.

## Figures and Tables

**Table 1:** Dough formulations with varying levels of sourdough addition.

Ingredients (g)	Sourdough Addition Level				
	0%	30%	50%	70%	100%
Wheat flour	1000	800	667	533	333
Sourdough	0	300	500	700	1000
Water	480	380	313	247	127
Semi-dry Yeast	10	10	10	10	10
White Sugar	30	30	30	30	30
Edible alkali	0.6	2.4	2.8	3.2	3.6

Note: Sourdough addition was calculated based on total flour weight (TFW), where TFW = flour in sourdough + flour added during the mixing stage.

**Table 2:** Scoring standard for sensory evaluation of frozen raw steamed stuffed buns.

Item	Score	Evaluation Criteria
Color	10	Surface appears white or milky white with distinct natural gloss (7–10 points)
		Surface slightly yellowish or creamy yellow, with weak gloss or slightly dull in local areas (4–7 points)
		Surface yellowish or dark, with dull gloss (1–4 points)
Surface Bubbles	10	Smooth surface, no bubbles (7–10 points)
		Moderately smooth surface, slight bubbles (4–7 points)
		Rough surface, significant bubbles (1–4 points)
Appearance	10	Plump, no shrinkage or collapse (7–10 points)
		Slightly poor plumpness, minor shrinkage or collapse (4–7 points)
		Severe shrinkage or collapse (1–4 points)
Elasticity	10	Recovers original shape quickly after pressing (7–10 points)
		Recovers original shape slowly after pressing (4–7 points)
		Fails to recover or recovers with difficulty after pressing (1–4 points)
Stickiness	10	Refreshing and non-sticky (7–10 points)
		Slightly sticky (4–7 points)
		Severely sticky (1–4 points)
Chewiness	10	Good chewiness, firm texture (7–10 points)
		Moderate chewiness, slightly hard or soft (4–7 points)
		Poor chewiness, overly hard or soft (1–4 points)
Softness/Hardness	10	Soft and elastic in the mouth (7–10 points)
		Slightly firm or somewhat too soft in the mouth (4–7 points)
		Dry, hard, and rough, or excessively soft and mushy in the mouth (1–4 points)

**Table 3:** Fermentation characteristics of dough with different sourdough addition levels.

Fermentation Characteristics	Sourdough Addition Level				
	0%	30%	50%	70%	100%
Hm (mm)	40.50 ± 0.85 ^e^	47.70 ± 0.99 ^d^	56.70 ± 1.70 ^c^	66.85 ± 0.49 ^b^	70.50 ± 1.98 ^a^
Tx (min)	49.50 ± 2.12 ^bc^	48.75 ± 5.30 ^c^	54.00 ± 2.12 ^bc^	57.50 ± 0.71 ^b^	70.25 ± 3.89 ^a^
Vt (mL)	1426.50 ± 24.75 ^bc^	1766.50 ± 125.16 ^a^	1411.00 ± 131.52 ^bc^	1503.50 ± 139.30 ^ab^	1147.50 ± 152.03 ^c^
Rc (%)	71.15 ± 0.35 ^c^	66.95 ± 1.63 ^d^	69.85 ± 0.07 ^c^	74.70 ± 0.42 ^b^	79.65 ± 0.21 ^a^

Note: Hm (mm), maximum height reached by the dough during fermentation; Tx (min), time at which dough porosity appears, i.e., the moment from which carbon dioxide gas begins to escape from the dough; Vt (mL), total volume of gas (CO_2_) produced during 3 h of fermentation; Rc (%), gas retention rate, referring to the percentage of gas (CO_2_) retained inside the dough relative to the total gas produced. Different lowercase letters indicate significant differences among samples for the same index, and the same convention applies to the tables below. Data were expressed as mean ± standard deviation. Means within the same column followed by different superscript lowercase letters represent significant difference (*p* < 0.05).

**Table 4:** Effects of different sourdough addition levels on the contents of organic acids and ethanol in dough during frozen storage.

	Sourdough Addition Level	Lactic Acid (mg/g)	Acetic Acid (mg/g)	Ethanol (mg/g)
Frozen for 1 days	0%	0.03 ± 0.00 ^d^	0.09 ± 0.00 ^b^	0.88 ± 0.06 ^b^
	30%	1.30 ± 0.01 ^c^	0.13 ± 0.00 ^a^	0.95 ± 0.02 ^b^
	50%	2.87 ± 0.06 ^b^	0.15 ± 0.01 ^a^	1.14 ± 0.02 ^a^
	70%	3.38 ± 0.47 ^b^	0.16 ± 0.02 ^a^	1.10 ± 0.04 ^a^
	100%	4.36 ± 0.11 ^a^	0.14 ± 0.01 ^a^	1.09 ± 0.06 ^a^
Frozen for 30 days	0%	N.D.	0.35 ± 0.01 ^a^	0.55 ± 0.01 ^b^
	30%	1.83 ± 0.14 ^d^	0.30 ± 0.02 ^b^	0.36 ± 0.01 ^c^
	50%	3.22 ± 0.17 ^c^	0.33 ± 0.00 ^a^	0.55 ± 0.03 ^b^
	70%	4.53 ± 0.05 ^b^	0.30 ± 0.00 ^b^	0.77 ± 0.01 ^a^
	100%	6.57 ± 0.09 ^a^	0.28 ± 0.01 ^b^	0.38 ± 0.04 ^c^

Note: ND means not detected. Data were expressed as mean ± standard deviation. Means within the same column followed by different superscript lowercase letters represent significant difference (*p* < 0.05).

**Table 5:** Effect of different sourdough addition levels on fermentable sugar content in dough during frozen storage.

	Sourdough Addition Level	Fructose Content (mg/g)	Glucose Content (mg/g)	Sucrose Content (mg/g)	Maltose Content (mg/g)	Total Content (mg/g)
Frozen for 1 days	0%	13.14 ± 0.21 ^a^	7.68 ± 0.13 ^b^	0.71 ± 0.09 ^d^	9.33 ± 0.01 ^e^	30.85 ± 0.27 ^e^
	30%	11.36 ± 0.34 ^b^	7.65 ± 0.19 ^b^	2.51 ± 0.11 ^b^	14.08 ± 0.30 ^d^	35.60 ± 0.94 ^d^
	50%	11.53 ± 0.01 ^b^	7.99 ± 0.11 ^b^	2.42 ± 0.07 ^b^	18.50 ± 0.02 ^c^	40.43 ± 0.02 ^c^
	70%	11.06 ± 0.25 ^b^	7.71 ± 0.30 ^b^	2.74 ± 0.03 ^a^	21.78 ± 0.30 ^b^	43.29 ± 0.88 ^b^
	100%	11.12 ± 0.24 ^b^	8.51 ± 0.06 ^a^	2.19 ± 0.02 ^c^	28.28 ± 0.30 ^a^	50.09 ± 0.59 ^a^
Frozen for 30 days	0%	11.85 ± 0.12 ^a^	7.98 ± 0.17 ^d^	0.66 ± 0.00 ^c^	9.43 ± 0.38 ^e^	29.92 ± 0.33 ^e^
	30%	9.92 ± 0.24 ^c^	7.99 ± 0.06 ^d^	2.62 ± 0.04 ^a^	14.38 ± 0.20 ^d^	34.91 ± 0.46 ^d^
	50%	9.91 ± 0.04 ^c^	8.44 ± 0.08 ^c^	2.56 ± 0.13 ^a^	18.01 ± 0.32 ^c^	38.91 ± 0.48 ^c^
	70%	10.63 ± 0.39 ^b^	9.04 ± 0.16 ^b^	2.75 ± 0.11 ^a^	22.35 ± 0.49 ^b^	44.76 ± 1.15 ^b^
	100%	10.50 ± 0.01 ^b^	9.63 ± 0.04 ^a^	2.26 ± 0.00 ^b^	28.84 ± 0.02 ^a^	51.23 ± 0.08 ^a^

Note: Data were expressed as mean ± standard deviation. Means within the same column followed by different superscript lowercase letters represent significant difference (*p* < 0.05).

**Table 6:** Effect of different sourdough addition levels on the molecular weight distribution of proteins in dough during frozen storage.

	Sourdough Addition Level	Molecular Weight Distribution of Proteins		
		SDS-P	SDS-M	SDS-I
Frozen for 1 days	0%	3.54%	8.58%	87.87%
	50%	12.13%	35.16%	52.72%
	100%	3.92%	59.99%	36.09%
Frozen for 30 days	0%	0.19%	32.17%	67.64%
	50%	0.15%	26.29%	73.56%
	100%	0.18%	27.76%	72.05%

Note: SDS-P: SDS-soluble polymers; SDS-M: SDS-soluble monomeric proteins; SDS-I: SDS-insoluble proteins.

## Data Availability

The data presented in this study are available on request from the corresponding authors (due to the ongoing nature of the research project).
